# Phase 1 Study of [^177^Lu]Lu-NeoB in Patients with Advanced Solid Tumors Overexpressing Gastrin-Releasing Peptide Receptor: Preliminary Safety and Dosimetry Results

**DOI:** 10.2967/jnumed.125.270411

**Published:** 2026-02

**Authors:** Erik S. Mittra, Lilja B. Solnes, Astrid A.M. van der Veldt, Jeffrey Wong, Luigi Aloj, Michael C. Heinrich, Christopher T. Chen, Steven P. Rowe, Tessa Brabander, Simon Pacey, Paola Aimone, Dhrubajyoti Pathak, Lars Blumenstein, Yongmin Liu, Andrei Iagaru

**Affiliations:** 1 Division of Molecular Imaging and Therapy, Department of Radiology, Oregon Health & Science University, Portland, Oregon;; 2 Division of Nuclear Medicine and Molecular Imaging, Russell H. Morgan Department of Radiology and Radiological Science, Johns Hopkins University School of Medicine, Baltimore, Maryland;; 3 Department of Radiology and Nuclear Medicine and Department Medical Oncology, Erasmus Medical Center, Rotterdam, The Netherlands;; 4 Department of Radiation Oncology, City of Hope National Cancer Center, Duarte, California;; 5 Department of Radiology, University of Cambridge, Cambridge, United Kingdom;; 6 Division of Hematology and Medical Oncology, Portland VA Health Care System and Oregon Health & Science University, Portland, Oregon;; 7 Division of Oncology, Department of Medicine, Stanford University School of Medicine, Stanford, California;; 8 Molecular Imaging and Therapeutics, Department of Radiology, University of North Carolina, Chapel Hill, North Carolina;; 9 Department of Radiology and Nuclear Medicine, Erasmus Medical Center, Rotterdam, The Netherlands;; 10 Department of Oncology, Clinical School, University of Cambridge, Cambridge, United Kingdom;; 11 Novartis Pharma AG, Basel, Switzerland;; 12 Novartis Biomedical Research, Basel, Switzerland;; 13 Novartis Pharma Co., Ltd., Beijing, China; and; 14 Division of Nuclear Medicine and Molecular Imaging, Department of Radiology, Stanford University, Stanford, California

**Keywords:** gastrin-releasing peptide receptor, [^177^Lu]Lu-NeoB, lutetium, radiopharmaceutical therapy, solid tumors

## Abstract

Gastrin-releasing peptide receptor (GRPR) is overexpressed in a range of tumor types, making it an attractive candidate for novel treatment approaches. NeoB binds to GRPR with high affinity and can be radiolabeled with ^68^Ga ([^68^Ga]Ga-NeoB) for imaging or ^177^Lu ([^177^Lu]Lu-NeoB, hereafter ^177^Lu-NeoB) for therapy, making it suitable for theranostics. **Methods:** NeoRay is a prospective, phase 1/2a, open-label, multicenter, first-in-human study of ^177^Lu-NeoB. Patients with selected advanced solid tumors with GRPR expression (confirmed by [^68^Ga]Ga-NeoB lesion uptake) were enrolled. Here, we report preliminary data (cutoff, April 29, 2024) from phase 1, which aimed to identify the maximum tolerated dose and/or recommended phase 2 dose of ^177^Lu-NeoB. Patients were scheduled to receive at least 3 cycles of ^177^Lu-NeoB at an interval of at least 6 wk. A Bayesian optimal interval design was used, with dose-escalation decisions based on dose-limiting toxicities (DLTs) during cycle 1 of each dose level. The primary endpoint was the incidence and nature of DLTs. Safety was assessed before and throughout each cycle. Dosimetry was assessed after the first administration. **Results:** Seventeen patients (median age, 65 y; 71% male) with advanced gastrointestinal stromal tumors, prostate cancer, glioblastoma, or breast cancer received ^177^Lu-NeoB activities of 1.85 GBq (cycle 1) and then 5.55 GBq (cycles 2–4) (*n* = 3), 9.25 GBq (*n* = 9), or 11.1 GBq (*n* = 5). Four DLTs were observed in 3 patients who received 11.1 GBq: grade 3 anemia (*n* = 2), grade 4 neurologic decline (*n* = 1), and grade 3 encephalopathy (*n* = 1). No DLTs were observed at lower administered activities. Overall, 4 of 17 patients (23.5%) had treatment-related adverse events of grade 3 or higher. Among patients with at least 1 evaluable dosimetry measurement (*n* = 16), the mean absorbed dose coefficient was 0.10 Gy/GBq (SD, 0.056 Gy/GBq) in the kidneys, 0.055 Gy/GBq (SD, 0.039 Gy/GBq) in the pancreas, and 0.018 Gy/GBq (SD, 0.0076 Gy/GBq) in the red marrow. **Conclusion:** ^177^Lu-NeoB has a favorable organ dosimetry profile in patients with advanced solid tumors expressing GRPR, with a large safety margin compared with accepted external beam radiation therapy thresholds for organ toxicity. The maximum tolerated dose of ^177^Lu-NeoB was identified as 9.25 GBq, and the recommended phase 2 dose for the phase 2a dose expansion is 9.25 GBq.

## Visual Abstract

**Figure:**
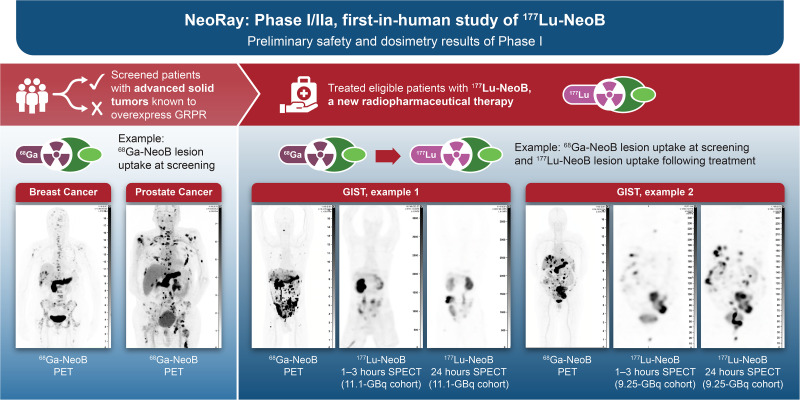

Gastrin-releasing peptide receptor (GRPR) is overexpressed in gastrointestinal stromal tumors (GISTs); glioblastomas; and breast, lung, and prostate cancer (1–5). Despite therapeutic advances, these cancers are still a frequent cause of death (6), and new treatment approaches are needed (2–5).

Tumor expression of GRPR makes it an attractive candidate for novel diagnostic and treatment approaches (7). NeoB is a novel nonactivating ligand of GRPR that binds with high affinity and specificity (8). NeoB can be radiolabeled with ^68^Ga for imaging or ^177^Lu for therapy, making it suitable for theranostics (9). In the phase 1/2a MITIGATE study, [^68^Ga]Ga-NeoB (hereafter ^68^Ga-NeoB) was considered to be a promising radiopharmaceutical for PET of tumor GRPR expression in patients with advanced GISTs, with strong tumor uptake observed in 50% of patients (10). Preliminary findings from the phase 2 NeoFIND study also support the utility of ^68^Ga-NeoB for the identification of GRPR-expressing tumors (11). Therapy with the radiopharmaceutical [^177^Lu]Lu-NeoB (hereafter ^177^Lu-NeoB) has demonstrated in vivo antitumor activity in mice bearing human GISTs (12).

NeoRay (NCT03872778) is a phase 1/2a, first-in-human study of ^177^Lu-NeoB (13). Its purpose is to characterize the safety, tolerability, pharmacokinetics, biodistribution, radiation dosimetry, and antitumor activity of ^177^Lu-NeoB in patients with selected advanced or metastatic solid tumors known to overexpress GRPR. The dose-escalation stage (phase 1) was designed to identify a safe and tolerated activity of ^177^Lu-NeoB for further evaluation in a larger patient population in the dose-expansion phase (phase 2a). This article reports preliminary phase 1 safety and dosimetry data; phase 2a is ongoing.

## MATERIALS AND METHODS

### Study Design and Patient Population

**FIGURE 1.**
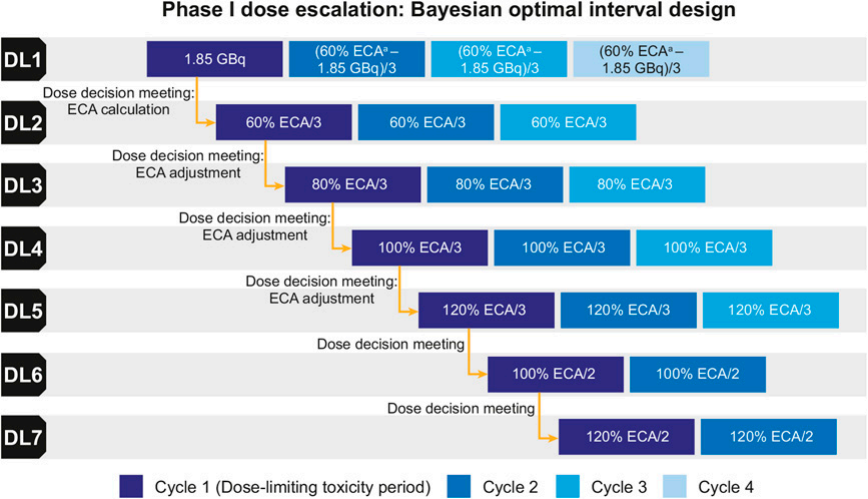
Study design. ^a^Maximum 5.55 GBq per cycle; additional cycles allowed in DL 1 or subsequent DLs if planned estimated cumulative activity (ECA) was not reached (sponsor medical monitor and investigator agreement required).

NeoRay is a prospective, phase 1/2a, open-label, multicenter study. Phase 1 followed a Bayesian optimal interval design (Fig. 1) (14,15), with dose-escalation decisions based on the occurrence of dose-limiting toxicities (DLTs) during cycle 1 of each dose level (DL).

In phase 1, patients were recruited from 6 centers in The Netherlands, U.K., and United States. Eligible patients were at least 18 y old and had advanced or metastatic breast, lung, or prostate cancer; GIST; or glioblastoma. In addition, they had at least 1 measurable tumor lesion that showed ^68^Ga-NeoB uptake greater than or equal to that of the spleen on PET/CT or PET/MRI, were not candidates for standard therapy, and had an Eastern Cooperative Oncology Group performance status of 2 or less. Further details and exclusion criteria are described in the supplemental materials (available at http://jnm.snmjournals.org).

NeoRay was approved by an institutional review board or independent ethics committee at each participating center and performed in accordance with the principles of the Declaration of Helsinki, the International Conference on Harmonisation Good Clinical Practice guidelines, and all applicable regulations. Written informed consent was obtained from patients before participation.

### Treatment

An intravenous injection of ^68^Ga-NeoB (recommended activity, 3 MBq/kg ± 10%; range, 150–250 MBq) was administered to confirm eligibility for ^177^Lu-NeoB treatment, with PET/CT or PET/MRI performed once 1–3 h later.

Patients eligible for ^177^Lu-NeoB received their first intravenous infusion within 6 wk of giving written informed consent. There were 7 provisional DLs, with patients due to receive at least 3 administrations at DLs 1–5 and 2 administrations at DLs 6–7; the interval between each administration was at least 6 wk (Fig. 1).

In DL 1, ^177^Lu-NeoB was initially administered at 1.85 GBq to establish tracer biodistribution, radiation dosimetry, and absorbed dose and residence time in critical organs. However, as it was considered unlikely that 1.85 GBq would deliver an absorbed dose to tumors that would translate into a clinical benefit, 5.55 GBq was selected for evaluation in the next 3 planned cycles of DL 1.

Dosimetry data obtained from 3 evaluable patients at cycle 1 of DL 1 were used to establish the maximum estimated cumulative activity for organs at risk (i.e., the red marrow, kidneys, and pancreas) for ^177^Lu-NeoB. The estimated cumulative activity was calculated from commonly accepted external beam radiation therapy (EBRT) thresholds for organ toxicity, that is, 2 Gy for red marrow (16), 23 Gy for kidneys (17,18), and 40 Gy for pancreas (17). The estimated cumulative activity was calculated to assess the maximum allowed cumulative administered activity, which could not cross EBRT thresholds. The maximum allowed cumulative administered activity informed the intrapatient dose escalation during DL 1 (up to a maximum of 5.55 GBq) and the administered activity for the next DL. At each DL, additional dosimetry data were obtained from at least the first 3 patients treated during cycle 1. These data, combined with dosimetry data from previous DLs, were used to adjust the estimated cumulative activity and, alongside safety data, to determine the next DL to be tested.

Administered activity adjustments were permitted if patients could not tolerate the ^177^Lu-NeoB protocol-specified treatment schedule, with a maximum of 1 reduction allowed.

### Endpoints

The primary objective of phase 1 was to identify the maximum tolerated dose (MTD) and/or recommended phase 2 dose (RP2D) of ^177^Lu-NeoB. The primary endpoint was the incidence and nature of DLTs. DLTs were defined as adverse events (AEs) or abnormal laboratory values meeting prespecified severity criteria that were not attributable to the disease or disease-related processes under investigation; were potentially related to ^177^Lu-NeoB; and occurred no more than 42 d after the first ^177^Lu-NeoB administration.

Secondary objectives and endpoints of phase 1 included characterizing the safety and tolerability of ^177^Lu-NeoB and assessing the biodistribution and radiation dosimetry (including time–activity curves and absorbed doses in critical organs [e.g., kidneys, red marrow, and pancreas]) of each ^177^Lu-NeoB DL. Results relating to other secondary objectives of phase 1—that is, assessing the pharmacokinetics and preliminary antitumor activity of ^177^Lu-NeoB and further characterizing the safety and tolerability of ^68^Ga-NeoB—are not reported in this article, which focuses on preliminary safety and dosimetry.

### Assessments

Safety was assessed during treatment (after each ^177^Lu-NeoB administration, every week during cycle 1, and every 2 wk during subsequent cycles) and during follow-up. AEs were coded using Medical Dictionary for Regulatory Activities version 26.1, with severity graded per Common Terminology Criteria for Adverse Events version 5.0.

Biodistribution and radiation dosimetry assessments included whole-body planar imaging (before administration and at 1–3, 6, 24, 48, 72, and 168 h) and SPECT/CT (at 1–3 and 24 h) after the first ^177^Lu-NeoB administration for at least the first 3 patients at each DL. Blood and urine samples were collected before, during, and at multiple time points after the first ^177^Lu-NeoB administration for at least the first 3 patients at each DL.

### Statistical Analyses

A Bayesian optimal interval design was implemented; therefore, a fixed cohort size was not required (14,15). However, at least 3 evaluable patients were enrolled at each DL. Dose-escalation decisions were based on the occurrence of DLTs during cycle 1 of each DL; further information is provided in the supplemental materials. Predefined assumptions were that the target DLT rate was 25% for the MTD, with 7 provisional DLs to be tested, and that there would be a maximum of 9 patients treated per DL and 36 patients treated overall.

All patients who received at least 1 administration of ^177^Lu-NeoB were included in the full analysis set. DLTs were assessed in the dose-determining set, that is, all patients from the full analysis set who either met the minimum exposure criterion (defined as those who received ≥90% of the planned administered activity of ^177^Lu-NeoB during cycle 1 and were observed for ≥42 d after the first administration) and had sufficient safety evaluations during cycle 1 to determine that a DLT did not occur, or discontinued earlier because of a DLT in cycle 1. Dosimetry was assessed in the dosimetry analysis set, that is, all patients with at least 1 evaluable dosimetry measurement.

Data were analyzed using SAS version 9.4 and were summarized for each DL using descriptive statistics. The supplemental materials include additional information about dosimetry analyses (19–28). Because of the descriptive nature of the study, no formal statistical comparisons were performed.

## RESULTS

### Patient Characteristics

**FIGURE 2.**
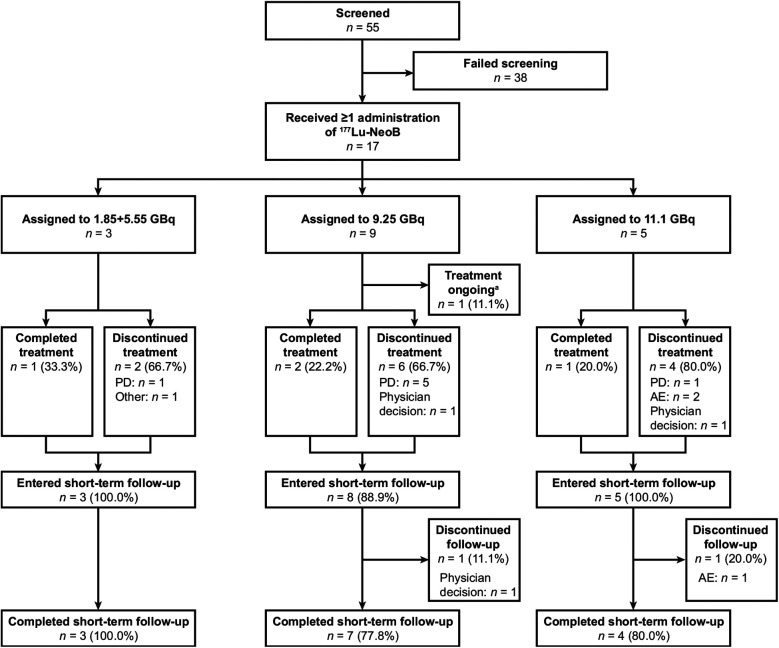
Patient disposition. ^a^Ongoing at data cutoff (April 29, 2024). PD = progressive disease.

Of 55 patients screened, 50 received ^68^Ga-NeoB (Fig. 2). Overall, 17 patients were enrolled and treated with ^177^Lu-NeoB between March 30, 2021, and April 18, 2023; demographics and baseline characteristics are reported in Table 1.

**TABLE 1.** Demographics and Baseline Characteristics (Full Analysis Set)

Variable	1.85 + 5.55-GBq DL, *n* = 3	9.25-GBq DL, *n* = 9	11.1-GBq DL, *n* = 5	All patients, *n* = 17
Age (y)	54.0 (22–70)	69.0 (19–77)	55.0 (49–70)	65.0 (19–77)
Sex				
Female	1 (33.3)	4 (44.4)	0	5 (29.4)
Male	2 (66.7)	5 (55.6)	5 (100.0)	12 (70.6)
Weight (kg)	72.2 (65.3–84.3)	79.9 (42.6–94.0)	91.3 (66.4–107.0)	79.9 (42.6–107.0)
Eastern Cooperative Oncology Group performance status				
0	2 (66.7)	4 (44.4)	3 (60.0)	9 (52.9)
1	1 (33.3)	5 (55.6)	2 (40.0)	8 (47.1)
Cancer type				
GIST	1 (33.3)	6 (66.7)	2 (40.0)	9 (52.9)
Breast	1 (33.3)	0	0	1 (5.9)
Glioblastoma	0	1 (11.1)	1 (20.0)	2 (11.8)
Prostate	1 (33.3)	2 (22.2)	2 (40.0)	5 (29.4)
Stage at study entry				
IV	3 (100.0)	8 (88.9)	5 (100.0)	16 (94.1)
Unknown	0	1 (11.1)	0	1 (5.9)
Months since initial diagnosis	64.99 (37.9–241.8)	84.44 (26.4–186.9)	67.25 (17.3–174.7)	75.89 (17.3–241.8)

Qualitative data are number and percentage; continuous data are median and range.

Examples of patient images after ^68^Ga-NeoB and ^177^Lu-NeoB administration are shown in the online graphical abstract.

### Treatment Exposure

At data cutoff (April 29, 2024), 1 patient was still receiving treatment, whereas 4 had completed treatment and 12 had discontinued treatment (of whom 7 discontinued because of progressive disease; Fig. 2). Fourteen patients completed the short-term follow-up period. Overall, 8 patients completed phase 1, 8 patients discontinued phase 1, and 1 patient was still receiving treatment. There was 1 death during the on-treatment period from active euthanasia.

Three patients enrolled in DL 1 received ^177^Lu-NeoB at 1.85 GBq (cycle 1) and then 5.55 GBq (cycles 2–4). Five patients received 11.1 GBq (DL 2; cycles 1–3), and 9 received 9.25 GBq (DL 3; cycles 1–3); no further DLs were required to identify the MTD or RP2D. Cumulative administered activities of 18.5, 27.75, and 33.3 GBq were planned for the 1.85 + 5.55-GBq, 9.25-GBq, and 11.1-GBq DLs, respectively (Table 2). The median duration of exposure to ^177^Lu-NeoB was 12.43 wk (range, 6.0–59.9 wk), and the median actual cumulative administered activity was 18.21 GBq (range, 7.2–73.3 GBq; mean, 21.1 GBq [SD, 15.5 GBq]) (Table 2).

**TABLE 2.** ^177^Lu-NeoB Exposure (Full Analysis Set)

Variable	1.85 + 5.55-GBq DL, *n* = 3	9.25-GBq DL, *n* = 9	11.1-GBq DL, *n* = 5	All patients, *n* = 17
Duration of exposure (wk)*	13.14 (13.1–41.3)	12.00 (6.0–59.9)	6.00 (6.0–18.3)	12.43 (6.0–59.9)
Planned cumulative administered activity (GBq)^†^	18.5	27.75	33.3	
Actual cumulative administered activity while on study treatment (GBq)^‡^	7.35 (7.2–28.4)	18.59 (9.1–73.3^§^)	11.29 (10.7–32.1)	18.21 (7.2–73.3)

* (date of last administered activity – date of first administered activity + 42 d)/7; data are median and range.

† For 4 cycles in 1.85 + 5.55-GBq DL and 3 cycles in other DLs.

‡ Data are median and range.

§ Patient who received maximum actual cumulative administered activity of 73.3 GBq had received 8 cycles of ^177^Lu-NeoB at time of data cutoff; despite this, no serious AEs were reported.

### DLTs

No DLTs were observed in the 1.85 + 5.55-GBq DL (cohort 1); therefore, the activity for the next DL was escalated to 11.1 GBq.

Four DLTs were observed in 3 of 5 patients who received 11.1 GBq at DL 2 (split across 2 cohorts based on DLT rate-based escalation/deescalation decisions: cohorts 2 [*n* = 4] and 4 [*n* = 1]) (Table 3); the activities for the subsequent cohorts (DL 3) were deescalated to 9.25 GBq, with activities of 11.1 GBq or higher ultimately eliminated from further study.

**TABLE 3.** DLTs (Dose-Determining Set)

DLT	11.1-GBq DL, *n* = 5	All patients, *n* = 17
Any	3 (60.0)	3 (17.6)
G3	2 (40.0)	2 (11.8)
G4	1 (20.0)	1 (5.9)
Blood and lymphatic disorders	2 (40.0)	2 (11.8)
Anemia G3	2 (40.0)	2 (11.8)
Nervous system disorders	2 (40.0)	2 (11.8)
Encephalopathy G3	1 (20.0)	1 (5.9)
Nervous system disorder G4	1 (20.0)	1 (5.9)

G = grade per Common Terminology Criteria for Adverse Events version 5.0. Data are number and percentage.

One patient with glioblastoma, for whom clinically significant neurologic abnormalities were observed during screening, experienced grade 2 nausea and grade 3 vomiting 4 d after initial administration of ^177^Lu-NeoB. This was followed by grade 3 neurologic decline (preferred term per Medical Dictionary for Regulatory Activities: nervous system disorder [DLT]) that necessitated hospitalization. Although nausea and vomiting were resolved, neurologic decline did not resolve despite dexamethasone treatment, leading to ^177^Lu-NeoB discontinuation. The DLT worsened (to grade 4) and was ongoing at the time of the patient’s death from active euthanasia.

One patient with prostate cancer and bone metastases, who had grade 2 anemia at baseline, developed grade 3 anemia (DLT) on day 37. The anemia began to resolve with supportive treatment; however, the patient later died of progressive disease.

One patient with GIST experienced grade 3 encephalopathy (DLT) on day 8 and grade 3 anemia (DLT) on day 10. Symptoms began around 1 wk after the first infusion; the patient initially had grade 2 vomiting and grade 3 hyponatremia, followed by grade 3 seizure on day 11. Benzodiazepine withdrawal syndrome was suspected to be a confounding factor, as treatment with diazepam (10 mg twice daily) was interrupted a few days after ^177^Lu-NeoB infusion.

As no DLTs were observed among the 9 patients who received 9.25 GBq at DL 3 (2 cohorts: cohorts 3 [*n* = 4] and 5 [*n* = 5]), sufficient data had been collected to fulfil the phase 1 objective of identifying the MTD and/or RP2D.

Further rationale for dose-escalation and dose-deescalation decisions is provided in the supplemental materials.

### Safety

A summary of AEs, including those relating to laboratory investigations, is provided in Supplemental Table 1. Overall, 15 of 17 patients (88.2%) had treatment-related AEs (TRAEs), including 4 of 17 patients (23.5%) with TRAEs of grade 3 or higher and 3 of 17 (17.6%) with serious TRAEs. TRAEs of grade 3 or higher included anemia (*n* = 2/17 [11.8%]) and 1 case each (*n* = 1/17 [5.9%]) of vomiting, encephalopathy, nervous system disorder, and pulmonary embolism. There was overlap between these TRAEs of grade 3 or higher and serious TRAEs: serious TRAEs included vomiting (*n* = 2/17 [11.8%]) and 1 case each (*n* = 1/17 [5.9%]) of encephalopathy, nervous system disorder, anemia, and pulmonary embolism. All TRAEs of grade 3 or higher and serious TRAEs were reported in the 11.1-GBq DL, except pulmonary embolism, which was reported for 1 patient in the 9.25-GBq DL on day 66 (grade 3). None of the serious AEs or TRAEs were fatal.

The most common AEs were fatigue, nausea, vomiting, peripheral edema, and abdominal pain, whereas the most common AE of grade 3 or higher was anemia. One AE led to treatment interruption (grade 1 platelet count decrease), and 1 AE led to treatment discontinuation (grade ≥3 neurologic decline); both occurred in the 11.1-GBq DL.

In terms of blood chemistry abnormalities of grade 3 or higher, 1 patient from the 9.25-GBq DL had increased γ-glutamyl transferase. In terms of hematology abnormalities of grade 3 or higher, 1 patient in the 1.85 + 5.55-GBq DL had lymphocytopenia, 1 patient in the 1.85 + 5.55-GBq DL had neutropenia, and 2 patients in the 11.1-GBq DL had decreased hemoglobin.

Electrocardiograms revealed a new absolute QT interval corrected for heart rate by the Fridericia formula of 451–480 ms in 2 of 17 patients (11.8%; 1 patient in the 1.85 + 5.55-GBq DL and 1 patient in the 11.1-GBq DL). An increase of 31–60 ms in this corrected interval was reported in 5 of 17 patients (29.4%; 3 in the 1.85 + 5.55-GBq DL and 2 in the 9.25-GBq DL), whereas a PR interval of more than 200 ms was reported in 2 of 17 patients (11.8%; both in the 9.25-GBq DL). No significant changes in vital signs were observed.

### Dosimetry

In the dosimetry analysis set (*n* = 16), the mean absorbed dose coefficients of ^177^Lu-NeoB in the kidneys, pancreas, and red marrow were 0.10 (SD, 0.056), 0.055 (SD, 0.039), and 0.018 (SD, 0.0076) Gy/GBq, respectively (Table 4). In all DLs, the mean projected cumulative absorbed doses (CADs) in the kidneys, pancreas, and red marrow were far below the EBRT thresholds (Table 4). Full dosimetry details are provided in Supplemental Table 2.

**TABLE 4.** Organ Dosimetry: Organs at Risk and Total Body (Dosimetry Analysis Set*)

	1.85 + 5.55-GBq DL, *n* = 3		9.25-GBq DL, *n* = 9^†^		11.1-GBq DL, *n* = 4*		All patients, *n* = 16*^†^
Location	Absorbed dose coefficient (Gy/GBq)	Projected CAD (Gy)	Absorbed dose coefficient (Gy/GBq)	Projected CAD (Gy)	Absorbed dose coefficient (Gy/GBq)	Projected CAD (Gy)	Absorbed dose coefficient (Gy/GBq)
Red marrow (by imaging)	0.018 (0.0037)	0.34 (0.068)	0.019 (0.011)	0.52 (0.30)	0.015 (0.0042)	0.51 (0.14)	0.018 (0.0076)
Kidney	0.14 (0.099)	2.5 (1.8)	0.11 (0.042)	3.0 (1.2)	0.066 (0.0058)	2.2 (0.19)	0.10 (0.056)
Pancreas	0.10 (0.044)	1.9 (0.82)	0.049 (0.025)	1.3 (0.69)	0.028 (0.0087)	0.95 (0.29)	0.055 (0.039)
Total body	0.018 (0.0059)	0.33 (0.11)	0.015 (0.0054)	0.43 (0.15)	0.0090 (0.0021)	0.30 (0.068)	0.014 (0.0056)

* One patient from 11.1-GBq DL was excluded because of poor imaging quality.

† For variables in this table, 3 patients from 9.25-GBq DL did not have evaluable measurement.

Projected CAD (Gy) = absorbed dose coefficient (Gy/GBq) × planned cumulative administered activity (GBq) over 3 cycles.

Data are mean and SD.

## DISCUSSION

In the dose-escalation phase of NeoRay, the first-in-human study of ^177^Lu-NeoB, 17 patients were enrolled who had advanced solid tumors with confirmed GRPR expression. No DLTs were identified when ^177^Lu-NeoB was administered at 1.85 + 5.55 or 9.25 GBq. However, DLTs of anemia, neurologic decline, and encephalopathy were reported among 3 patients who received 11.1 GBq. Of note, the patient who experienced neurologic decline had glioblastoma, and clinically significant neurologic abnormalities were already present during screening, which may be an alternative explanation for the occurrence of this DLT. Additionally, for the DLT of encephalopathy, benzodiazepine withdrawal syndrome was suspected to be a confounding factor, as treatment with high doses of diazepam was interrupted shortly after ^177^Lu-NeoB infusion.

On the basis of these DLT findings, the MTD of ^177^Lu-NeoB was identified as 9.25 GBq, and this was declared to be the RP2D. The phase 2a dose expansion will assess the pharmacokinetics, dosimetry, and antitumor activity of the RP2D when administered for at least 2–3 cycles (depending on cohort) every 6 wk.

Our data provide initial insights into the safety profile of ^177^Lu-NeoB. Although AEs were recorded for all patients, only 1 TRAE of grade 3 or higher was reported among patients treated with the RP2D (9.25 GBq). This pulmonary embolism was defined as a serious TRAE but not considered a DLT because it occurred during cycle 2, that is, outside the DLT evaluation period. Of note, pulmonary embolisms are a common confounding factor in patients with advanced cancers. By comparison, serious TRAEs of grade 3 or higher occurred in a greater proportion of the 11.1-GBq DL. This included vomiting in addition to the 3 DLTs (anemia, neurologic decline, and encephalopathy). These findings suggest a more favorable safety profile with 9.25 versus 11.1 GBq.

A preclinical biodistribution study of ^177^Lu-NeoB in healthy mice found that the organs with the highest absorbed dose of ^177^Lu-NeoB were the kidneys, liver, and pancreas (29). By comparison, the highest projected CAD of ^177^Lu-NeoB in the NeoRay dose-escalation phase was in the kidneys (followed by the pancreas), although limited radiation was absorbed in other organs. Given that the absorbed doses in other organs ranged from low to very low, no correlation could be made with the observed AEs. Compared with EBRT thresholds, the organ dosimetry profile of ^177^Lu-NeoB displayed a large safety margin, and projected CADs indicate that the risk for late radiation-induced toxicities is very low. As such, on the basis of our preliminary dosimetry data, ^177^Lu-NeoB appears to have a favorable organ dosimetry profile; however, this will be further characterized in the phase 2a dose expansion study.

Limitations of this dose-escalation study include the small sample size; however, a small sample is typical for phase 1, first-in-human studies. Additionally, a high proportion of patients who received ^68^Ga-NeoB (*n* = 33/50 [66%]) were excluded from phase 1, primarily because of insufficient ^68^Ga-NeoB uptake. As a result, the relevant inclusion criterion was modified after study commencement (as described in the supplemental materials); therefore, screening data from phase 2 will provide greater insights on the optimal selection criteria for theranostic use of NeoB. Finally, further clinical evaluation is required to characterize the overall safety risk of ^177^Lu-NeoB.

## CONCLUSION

During the NeoRay phase 1 dose escalation, DLTs occurred only at the highest tested administered activity of ^177^Lu-NeoB (11.1 GBq). The results demonstrated a favorable organ dosimetry profile in patients with selected advanced solid tumors known to overexpress GRPR, with a large safety margin compared with EBRT thresholds, even at high administered activities. The RP2D to be further evaluated in the dose-expansion phase is 9.25 GBq.

## DISCLOSURE

NeoRay was funded by Advanced Accelerator Applications, a Novartis company. Luigi Aloj and Simon Pacey’s research was conducted using facilities supported by the NIHR Cambridge BRC (NIHR203312), NIHR Cambridge CRF, the Cambridge ECMC (C96/A25177/RG86728), Cancer Research UK Cambridge Center (C9685/A25117), and the Cambridge Human Research Tissue Bank, which is supported by the NIHR Cambridge BRC and BRC-JCB Hub services (BRC-1215-20014). The views expressed are those of the authors and not necessarily those of the NHS, the NIHR, or the Department of Health and Social Care. Erik Mittra has institutional research grants from Curium, ITM, Novartis, and RayzeBio and is a consultant for or receives honoraria from Clarity, Curium, and Sanofi. Lilja Solnes has grants from Cellectar, Novartis, and Perspectives Therapeutics and royalties from Elsevier. Astrid van der Veldt is a consultant (fees paid to the institute) for BMS, Eisai, GE HealthCare, Genentech, Ipsen, MSD, Novartis, Pierre Fabre, Pfizer, Roche, and Sanofi and receives travel fees from Ipsen. Jeffrey Wong has grants from Accuray, Blue Earth Diagnostics, NIH, Reflexion, and Varina and receives travel fees from Accuray. Luigi Aloj receives grants from Novartis, receives honoraria from SIRtex, is involved in clinical trials for Bayer and Novartis, and receives travel fees from Novartis. Michael Heinrich receives grants from the Department of Veterans Affairs (1 I01 BX005358-01A1), NIH National Cancer Institute (U01CA278470-01A1), and the GIST Cancer Research Fund (philanthropic donations) and is a consultant for Cogent, Deciphera Pharmaceuticals, IDRX, New Bay Pharmaceuticals, Novartis, and SAB (von Pfeffel Pharmaceuticals); additionally, he received funding from the NIH National Cancer Institute (1 R21 CA263400-01) while the NeoRay study was ongoing. Christopher Chen receives grants from ADC Therapeutics, Bolt Biotherapeutics, D3 Bio, Eli Lilly, Genentech-Roche, Gilead Sciences, GSK, Kinnate Biopharma, Mersana, ORIC Pharmaceuticals, Palleon Pharmaceuticals, Pionyr Therapeutics, Rain Oncology, Revolution Medicines, Seagen, Tango Therapeutics, and Takeda and is a consultant or on advisory boards for Johnson & Johnson, Mubadala Capital, Radionetics Oncology, and Tango Therapeutics. Steven Rowe is a consultant for Blue Earth, Lantheus, and Telix and receives honoraria from Lantheus. Tessa Brabander has received research support and speaker fees from AAA/Novartis (paid to her institute). Simon Pacey receives grants and travel frees from AstraZeneca (paid to his institute). Paola Aimone, Dhrubajyoti Pathak, and Lars Blumenstein are full-time employees of Novartis and own Novartis stock and stock options. Yongmin Liu is a full-time employee of Novartis. Andrei Iagaru receives grants from GE HealthCare and Novartis Pharmaceuticals; is a consultant for GE HealthCare, Lilly, Novartis Pharmaceuticals, Progenics Pharmaceuticals, and Telix; is on scientific advisory boards for Alpha-9 Theranostics, Clarity Pharmaceuticals, Nucleus RadioPharma, and Radionetics Oncology; and is on a study steering committee for Novartis Pharmaceuticals. Funding for medical writing support was provided by Advanced Accelerator Applications, a Novartis company. No other potential conflict of interest relevant to this article was reported.

## Notes

KEY POINTS **QUESTION:** What are the preliminary safety and dosimetry findings from the dose-escalation phase of NeoRay (the first-in-human phase 1/2a study of ^177^Lu-NeoB)? **PERTINENT FINDINGS:** Seventeen patients with advanced solid tumors expressing GRPR were treated with ^177^Lu-NeoB at 1.85 + 5.55 GBq (*n* = 3), 9.25 GBq (*n* = 9), or 11.1 GBq (*n* = 5). Four patients had TRAEs of grade 3 or higher. The MTD and RP2D were identified as 9.25 GBq based on 4 DLTs reported among 3 patients from the 11.1-GBq DL. Preliminary dosimetry data indicated a favorable organ dosimetry profile. **IMPLICATIONS FOR PATIENT CARE:** Findings support the ongoing clinical development of ^177^Lu-NeoB as a therapeutic agent for patients with advanced solid tumors known to overexpress GRPR.
